# A dataset of Natural Gas and Liquid Level for Oil Field Production Prediction in China

**DOI:** 10.1038/s41597-025-05309-w

**Published:** 2025-06-23

**Authors:** Yanlei Wang, Jian Lian, Chengjiang Li

**Affiliations:** 1 Shandong University of Political Science and Law, Jinan, 250357 China; 2 School of Intelligence Engineering, Shandong Management University, No.3500 Dingxiang Road, Jinan, 250357 Shandong China; 3 Jinan Campus, Shandong University of Science and Technology, No.17 Shenglizhuang Road, Jinan, 250351 Shandong China

**Keywords:** Natural gas, Power distribution

## Abstract

Natural gas, a critical resource for national economies and public welfare, plays a significant role in the energy sector. The efficient production of natural gas is often hindered by the presence of formation water, which can adversely affect well productivity and operational efficiency. Accurate prediction of natural gas production and estimation of associated fluid accumulation are therefore paramount for optimizing extraction processes. This study introduces a dataset compiled from multiple wells in the Inner Mongolia region, primarily targeting the Shanxi Formation, with the aim of enhancing the predictive accuracy of natural gas production and associated fluid accumulation. The dataset, spanning from March 17, 2010, to April 15, 2024, includes detailed records of wellhead pressure, casing pressure, daily methanol injection, and cumulative production volumes of gas, water, and oil. By analyzing these parameters, we can identify trends and anomalies over time, which are essential for refining production strategies and mitigating the impact of fluid accumulation on gas wells.

## Background & Summary

Natural gas, a vital component of the global energy mix, is essential for both industrial development and domestic use^1^. The production of natural gas is a complex process that involves the extraction of hydrocarbons from subterranean reservoirs^2^. In the Inner Mongolia region, the Shanxi Formation has been identified as a significant reservoir for natural gas, making it a focal point for our study^3^. However, the presence of formation water, commonly referred to as “fluid accumulation” or “Jiye” in Chinese, can impede gas flow, reduce well productivity, and increase operational costs^4^. The accurate prediction of natural gas production and the estimation of associated fluid accumulation are crucial for the efficient management of gas fields.

The ability to forecast natural gas yields and the concurrent accumulation of formation water is not only technically challenging but also economically significant^5^. Accurate predictions enable oil and gas companies to plan production more effectively, optimize resource allocation, and manage the environmental impacts of their operations. However, the complexity of subsurface conditions and the variability in reservoir characteristics make this a difficult task^6^. It is against this backdrop that the importance of our dataset becomes apparent.

This study presents a compiled dataset from multiple wells in the Inner Mongolia region, with a primary focus on the Shanxi Formation. The dataset encompasses a wide range of parameters, including wellhead pressure^7^, casing pressure^8^, daily methanol injection volumes, and cumulative production of gas, water, and oil. Spanning from March 17, 2010, to April 25, 2024, this dataset provides a detailed account of the operational history of these wells, capturing the dynamic interplay between natural gas production and fluid accumulation. The primary objective of this dataset is to enhance the accuracy of predictions regarding natural gas production and associated fluid accumulation. By providing a comprehensive and standardized dataset, we aim to facilitate advanced analytical techniques and modeling approaches that can improve the understanding of production dynamics in gas fields. This, in turn, can lead to more informed decision-making in the planning and management of natural gas production, with positive ramifications for the transportation, marketing, and other industries dependent on a stable supply of natural gas.

To our best knowledge, the dataset presented in this study is a valuable resource for researchers, engineers, and policymakers. It not only contributes to the scientific understanding of gas field operations but also serves as a foundation for developing more effective strategies for the sustainable extraction of natural gas resources.

## Methods

### Data Collection

Data was collected from a cohort of wells in the Inner Mongolia region. These wells are outfitted with state-of-the-art sensors^9^ capable of precisely measuring wellhead pressure, casing pressure, and the flow rate of gas. The sensors undergo regular calibration to guarantee the accuracy of the measurements. The data collection process was designed to minimize errors and safeguard the integrity of the data^10^. The data was collected on a daily basis to capture short-term variations in production parameters.

To ensure data quality, a comprehensive suite of quality control measures was implemented^11^. Outlier detection algorithms were utilized to identify and rectify any abnormal data points. Data integrity checks were performed to confirm that all requisite parameters were recorded and that there were no missing values. In cases where missing values were detected, the interpolation methods^12^ were employed based on the characteristics of the data and the production process.

### Data Pre-processing

The gathered data went through the following preprocessing procedures. Initially, the missing values within the dataset were detected and addressed. When the missing values were scattered and the time-series characteristic of the data needed to be maintained, interpolation methods were taken into account. Given that ${y}_{{t}_{i-1}}$ and ${y}_{{t}_{i+1}}$ are the known values neighboring the missing value ${y}_{{t}_{i}}$, the linearly interpolated value ${\widehat{y}}_{{t}_{i}}$ is computed as Equation (1):

1 $${\widehat{y}}_{{t}_{i}}={y}_{{t}_{i-1}}+\frac{({y}_{{t}_{i+1}}-{y}_{{t}_{i-1}})}{({t}_{i+1}-{t}_{i-1})}({t}_{i}-{t}_{i-1}),$$

Subsequently, the data was normalized so as to make all features have a comparable scale. This was accomplished by employing standard normalization methods like min-max scaling. Min-max scaling converts the data to a fixed interval [0, 1]. For a feature *x*, the scaled value *x*_*scaled*_ is determined by Equation (2):

2 $${x}_{scaled}=\frac{x-{x}_{min}}{{x}_{max}-{x}_{min}}$$

Here, *x*_*min*_ and *x*_*max*_ represent the minimum and maximum values of the feature *x* in the dataset respectively.

### Data Annotation

In this dataset, each data record was labeled with the corresponding well ID and date. Supplementary annotations were provided to denote any special events or operational changes that might have influenced the production parameters, such as equipment maintenance or alterations in injection strategies.

To ensure the consistency and accuracy of the annotations, a team of seasoned engineers and data analysts reviewed and verified the annotated data. Any discrepancies or ambiguities were resolved through in-depth discussions and consultations with field experts.

The dataset is organized in a structured manner and is publicly accessible for research purposes. It is partitioned into training and testing subsets using a stratified sampling approach to ensure that each subset contains a representative sample of the data^13^.

### Dataset statistics

In general, Table 1 provides a comprehensive and detailed overview of the key parameters and data related to the natural gas production process. It encompasses various crucial aspects such as production time, well characteristics, output volumes, pressure and temperature conditions, and injection volumes. This table serves as a vital tool for in-depth analysis and understanding of the complex dynamics and performance of the gas production system. By presenting these data in a systematic manner, it enables us to identify patterns, trends, and potential correlations that are essential for making informed decisions and formulating effective strategies to optimize the production process and enhance overall productivity.

**Table 1:** Detailed overview of the data fields related to the natural gas production.

Field Name	Data Type	Description
Date	Datetime	Accurately record the time of each piece of data for analyzing the time trend and periodic changes in the production process.
Well ID	String	Uniquely identify different production wells to facilitate separate analysis and comparison of production data for each well.
Layer	String	Describe the gas production layer, helping to understand the production characteristics of different layers.
Days of production	Int	Reflect the production time length of each well from the start of production to the statistical moment to assess the well’s production stability and maturity.
Production hours	Int	Represent the daily production duration for analyzing production efficiency.
Total production hours	Int	Accumulate the total production time from the start of production to the current moment to understand the overall production contribution of the well.
Production manner	String	Record different production operation modes, such as normal production, intermittent production, or maintenance production, which is of significant importance for studying the impact of production methods on output.
Gas volume	Float	Record the daily natural gas output in standard units of 10^4^*m*^3^, which is one of the key indicators for evaluating the gas production capacity.
Water volume	Float	Record the daily water output to analyze the impact of water phase changes in the production process on gas production −*m*^3^.
Oil volume	Float	Reflect the daily oil output for a comprehensive evaluation of the overall benefit of oil and gas production −*m*^3^.
Total gas production	Float	Accumulate the cumulative natural gas output from the start of production to the current moment to show the long-term production trend −10^4^*m*^3^.
Total water	Float	Accumulate the cumulative water output from the start of production to the current moment to reflect the water phase production situation −*m*^3^.
Total oil production	Float	Accumulate the cumulative oil output from the start of production to the current moment for a comprehensive assessment of the overall benefit of oil and gas production −*m*^3^.
Wellhead pressure	Float	May initially be recorded as a string value for pressure and can be converted to a numerical value after processing for analyzing the impact of wellhead pressure on production.
Casing pressure	Float	Record the inlet pressure, which is important for evaluating the pressure condition of the entire production system *Mpa*.
Daily methanol injection volume	Float	Record the daily methanol injection volume, which has a certain impact on regulating the fluid properties in the production process -*L*.
Monthly cumulative methanol injection volume	Float	Accumulate the monthly cumulative methanol injection volume to analyze the long-term effect of the methanol injection strategy -*L*.
Comparison of casing pressure with the previous day	Float	Record the comparison of casing pressure with the previous day to help analyze the pressure change trend -(*Mpa*).
Stage rate of pressure drop in casing	String	May be various types of data that require further analysis to determine for describing the pressure drop rate in a specific stage -(*Mpa*/*d*).
Memo	String	Provide supplementary explanations and notes regarding the production data, which may contain special circumstances, operation adjustments, and other information.

In this dataset, there is also data related to fluid accumulation. Since fluid accumulation does not occur every day and its occurrence rate is relatively low, it is stored separately in the dataset. The main stored contents are the time when fluid accumulation occurs and the degree of fluid accumulation. This degree is classified into the following types, including absence, minimal, mild, moderate, and severe. In this study, the classification of fluid accumulation severity in the natural gas production process is determined according to the scores given by on-site engineers. In total, Table 2 presents the key statistics of the dataset:

**Table 2:** Dataset Statistics. F, A, B, C, D, E denote fluid accumulation, absence, minimal, mild, moderate, and severe, respectively.

Well ID	No. Samples	Start of Date	End of Date	No. F	No. A	No. B	No. C	No. D	No. E
1	892	2021-11-06	2024-04-15	6	0	1	3	2	0
2	2320	2017-12-09	2024-04-15	6	0	1	3	2	0
3	2321	2017-12-08	2024-04-15	8	1	1	2	3	1
4	1578	2019-12-21	2024-04-15	1	0	1	0	0	0
5	1576	2019-12-23	2024-04-15	3	0	1	1	0	1
6	2328	2017-12-01	2024-04-15	6	1	1	2	2	0
7	4211	2012-10-05	2024-04-15	9	1	1	3	2	2
8	177	2023-10-22	2024-04-15	2	0	1	1	0	0
9	1321	2020-09-03	2024-04-15	4	0	1	2	1	0
10	3404	2014-12-21	2024-04-15	13	1	0	5	3	4
11	180	2023-10-23	2024-04-19	1	0	1	0	0	0
12	181	2023-10-22	2024-04-19	1	0	1	0	0	0
13	1262	2020-11-05	2024-04-19	4	0	0	2	2	0
14	1325	2020-09-03	2024-04-19	5	1	0	2	2	0
15	3408	2014-12-21	2024-04-19	8	1	0	1	3	3
16	1450	2020-05-01	2024-04-19	2	0	1	0	0	1
17	2743	2016-10-16	2024-04-19	6	0	1	3	1	1
18	5240	2020-12-15	2024-04-19	10	1	1	2	4	2
19	1268	2020-10-30	2024-04-19	1	0	0	0	0	1
20	1454	2020-04-27	2024-04-19	3	0	1	0	1	1
21	5154	2010-03-17	2024-04-25	6	1	0	1	2	2
22	2748	2016-10-17	2024-04-25	10	0	1	4	4	1
23	1829	2019-04-24	2024-04-25	11	1	0	4	3	3
24	1829	2019-04-24	2024-04-25	7	1	0	3	3	0
25	1243	2020-11-30	2024-04-25	6	0	1	2	2	1
26	1264	2020-11-09	2024-04-25	3	0	1	1	1	0
27	1822	2019-05-01	2024-04-25	2	0	1	1	0	0
28	1829	2019-04-24	2024-04-25	5	0	1	2	2	0
29	1830	2019-04-23	2024-04-25	4	0	1	1	1	1
30	3083	2015-11-17	2024-04-25	2	0	1	1	0	0
Total	61270	—	—	155	10	22	52	46	25

### LSTM Architecture

**Fig. 1:**
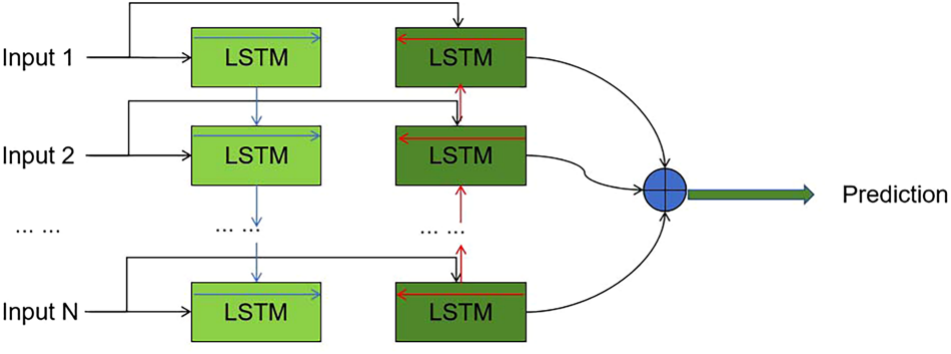
The LSTM architecture introduced in this study.

The Bidirectional Long Short-Term Memory (Bi-LSTM)^14^ network utilized in this study consists of an input layer, multiple hidden layers of LSTM cells, and an output layer (as shown in Fig. 1). The input layer takes in the time-series data related to wellhead pressure, casing pressure, and daily methanol injection. Each LSTM cell in the hidden layers contains a memory cell and three gates: the input gate, the forget gate^15^, and the output gate. The input gate controls the flow of new information into the memory cell, the forget gate determines what information to discard from the cell’s previous state, and the output gate decides what information to output for the current time step. This architecture allows the model to capture both the forward and backward temporal dependencies in the data, making it well-suited for handling the sequential nature of the natural gas production and fluid accumulation data.

For the hyperparameters of the introduced LSTM model, a batch size of 32 was chosen. The Adam optimizer was adopted, with a learning rate set to 1e-9. The model depth was specified as 8, the dropout rate was set at 0.5, and the number of epochs was fixed at 200.

### Model Performance Results

In this study, the evaluation metrics utilized are the Root Mean Squared Error (RMSE) and the Mean Absolute Percentage Error (MAPE). The RMSE formula is presented as Equation (3):

3 $$RMSE=\sqrt{\frac{1}{n}\mathop{\sum }\limits_{i=1}^{n}{({y}_{i}-{\widehat{y}}_{i})}^{2}},$$

where n stands for the overall number of samples or data points. *y*_*i*_ indicates the actual observed value of the i-th sample, and ${\widehat{y}}_{i}$ represents the corresponding predicted value. The formula for MAPE is given by Equation (4):

4 $$MAPE=\frac{1}{n}\mathop{\sum }\limits_{i=1}^{n}\left|\frac{{y}_{i}-\widehat{y}i}{yi}\right|\times 100$$

where n refers to the number of samples.

In the experiments, 80% of the records in the dataset is taken as the training set, and the remaining 20% records are taken as the testing set. And the performance of the introduced model on this dataset is presented in Table 3:

**Table 3:** Bi-LSTM Model Performance Results.

Metric	Performance
RMSE (Gas Production Prediction)	0.0865
MAPE (Gas Production Prediction)	0.0785

These metrics provide a comprehensive assessment of the Bi-LSTM model’s effectiveness in handling the prediction and detection tasks based on the collected dataset. Moreover, this study has also used random forest (RF), decision tree (DT), and support vector machine (SVM) as the baseline models.

In addition, a permutation feature importance analysis was conducted on the proposed model. For features like wellhead pressure and casing pressure, their values were shuffled in the test dataset and the changes in RMSE and MAPE were observed. Table 4 shows that shuffling wellhead pressure and casing pressure led to a significant increase in both RMSE and MAPE, indicating its high importance for the model’s performance. Daily methanol injection volume has a relatively minor effect.

**Table 4:** Feature Impact Analysis.

Feature	RMSE	MAPE
Without Wellhead Pressure	0.2278	0.1772
Without Casing Pressure	0.1436	0.1539
Without Wellhead Pressure and Casing Pressure	0.1247	0.1242

## Data Records

The proposed dataset is publicly available on the ScienceDB^16^ platform to facilitate researcher access. It consists of 31 documents, thirty of them dedicated to storing the natural gas production and liquid level samples for each well in the form of .xlsx format and the other one containing the wells’ statistical information in the form of .xlsx.

The sample file of each well consists of two spreadsheets, one spreadsheet contains the daily natural gas production data and the other spreadsheet contains the liquid level data. And the fields in the first spreadsheet are provided in Table 1. The fields in the second spreadsheet are provided in Table 2.

## Technical Validation

**Fig. 2:**
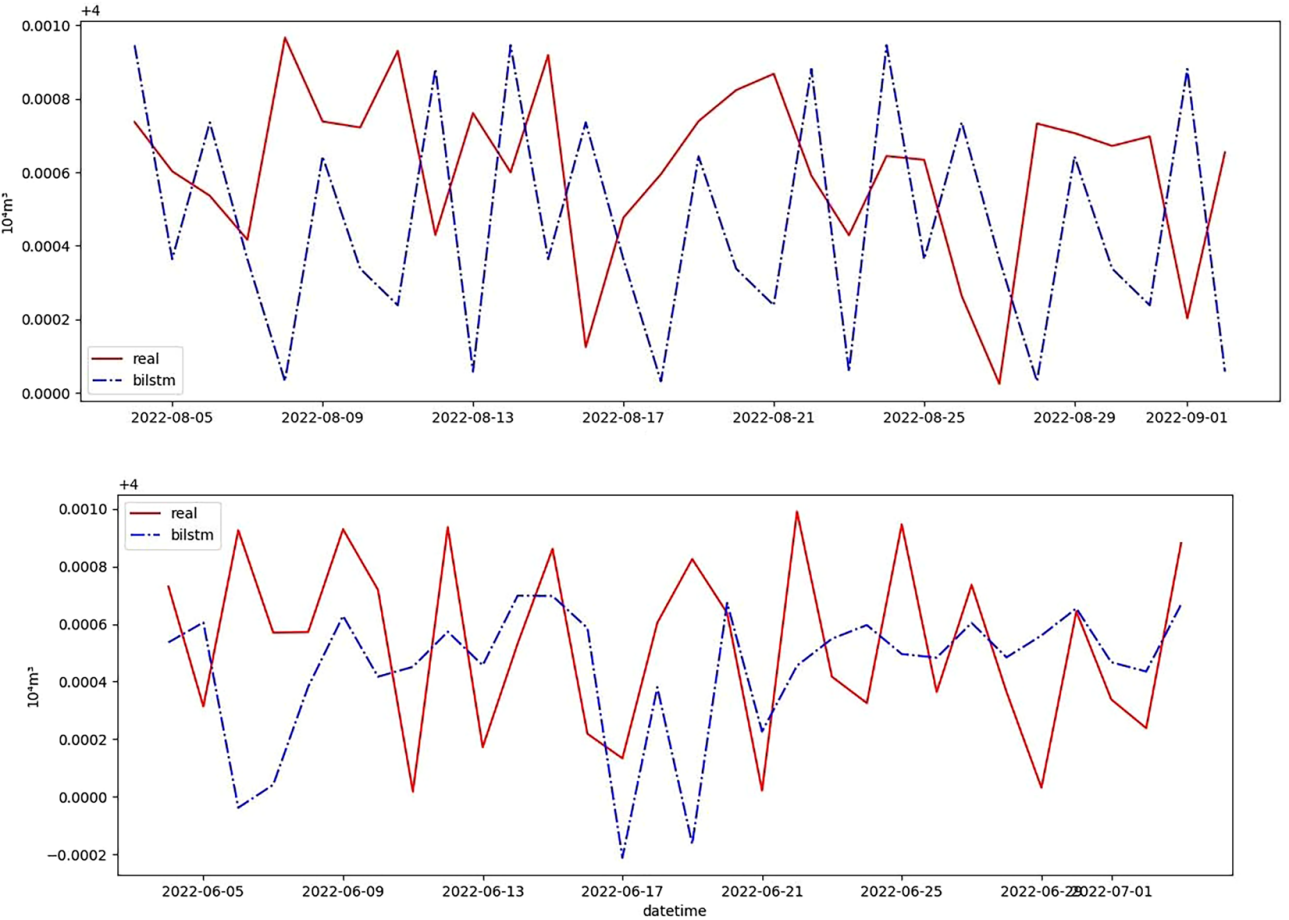
Comparison between the proposed model and the real natural gas production data records across different wells and time periods.

To validate the dataset’s quality and reliability, a series of tests were conducted. Data consistency checks were performed to ensure that the data was consistent across different wells and time periods, as shown in Fig. 2. Completeness checks were carried out to verify that all required data was present.

A separate validation process was dedicated to the annotations. The annotated data was randomly sampled and cross-checked by an independent team of experts who were not involved in the initial annotation process. They verified the accuracy of the well ID, date, time, and event annotations. In cases where errors or inconsistencies were found, the original annotation team was notified and the necessary corrections were made. Additionally, statistical analyses were performed on the annotations to check for any biases or patterns that could potentially affect the analysis. For example, the distribution of special events across different wells and time periods was examined to ensure that there was no over- or under-representation. This comprehensive annotation validation process further enhanced the reliability and usability of the dataset.

## Usage Notes

### Potential applications

The dataset presented in this study holds significant potential for various applications within the natural gas production domain. It can be utilized for the development and training of advanced machine learning and artificial intelligence models focused on natural gas production prediction. By leveraging the detailed records of wellhead pressure, casing pressure, daily methanol injection, gas production volume, and fluid accumulation information, researchers and industry practitioners can enhance the accuracy of their predictive models. This, in turn, enables more efficient production planning, allowing for optimized resource allocation and timely decision-making regarding well operations.

Furthermore, the dataset can also be used in the field of reservoir engineering for the analysis of reservoir behavior and the assessment of the impact of fluid accumulation on gas production. Engineers can use the data to study the correlations between different parameters and gain a deeper understanding of the underlying physical processes. This knowledge can then be applied to design more effective production strategies and to implement appropriate measures for mitigating the negative effects of fluid accumulation.

### Limitations

Despite its potential applications, the dataset has certain limitations. One of the primary limitations is the geographical scope of the data collection. The dataset is sourced from multiple wells in the Inner Mongolia region, specifically targeting the Shanxi Formation. This limited geographical area may restrict the generalizability of the models trained using this dataset to other regions with different geological characteristics. The reservoir properties, fluid compositions, and production behaviors can vary significantly from one region to another, and thus, the models developed based on this dataset may not perform equally well in other locations.

Another limitation is the potential presence of measurement errors and uncertainties in the data. Although efforts have been made to ensure data quality through calibration of sensors and implementation of quality control measures, there is still a possibility of errors in the recorded values. These errors could affect the accuracy of the models trained with the dataset and lead to less reliable predictions and analyses.

## Data Availability

The code used for data processing, analysis, and model development is available on Gitee at https://gitee.com/practicing-swordsmanship/jian-lian/tree/master. The code is comprehensively documented, enabling other researchers to reproduce the analysis and build upon the work. It encompasses functions for data cleaning, feature engineering, model training, and evaluation.
